# CrisPam: SNP-Derived PAM Analysis Tool for Allele-Specific Targeting of Genetic Variants Using CRISPR-Cas Systems

**DOI:** 10.3389/fgene.2020.00851

**Published:** 2020-08-18

**Authors:** Roy Rabinowitz, Shiri Almog, Roy Darnell, Daniel Offen

**Affiliations:** ^1^Department of Human Molecular Genetics and Biochemistry, Sackler School of Medicine, Tel Aviv University, Tel Aviv, Israel; ^2^Felsenstein Medical Research Center, Tel Aviv University, Tel Aviv, Israel; ^3^Sagol School of Neuroscience, Tel Aviv University, Tel Aviv, Israel

**Keywords:** CrisPam, allele-specific, clustered regularly interspaced short palindromic repeats, single-nucleotide polymorphism-derived protospacer adjacent motif, guide RNA design

## Abstract

Clustered regularly interspaced short palindromic repeats (CRISPR) is a promising novel technology that holds the potential of treating genetic diseases. Safety and specificity of the treatment are to be further studied and developed prior to implementation of the technology into the clinic. The guide-RNA (gRNA) allows precise position-specific DNA targeting, although it may tolerate small changes such as point mutations. The permissive nature of the CRISPR-Cas system makes allele-specific targeting a challenging goal. Hence, an allele-specific targeting approach is in need for future treatments of heterozygous patients suffering from diseases caused by dominant negative mutations. The single-nucleotide polymorphism (SNP)-derived protospacer adjacent motif (PAM) approach allows highly allele-specific DNA cleavage due to the existence of a novel PAM sequence only at the target allele. Here, we present CrisPam, a computational tool that detects PAMs within the variant allele for allele-specific targeting by CRISPR-Cas systems. The algorithm scans the sequences and attempts to identify the generation of multiple PAMs for a given reference sequence and its variations. A successful result is such that at least a single PAM is generated by the variation nucleotide. Since the PAM is present within the variant allele only, the Cas enzyme will bind the variant allele exclusively. Analyzing a dataset of human pathogenic point mutations revealed that 90% of the analyzed mutations generated at least a single PAM. Thus, the SNP-derived PAM approach is ideal for targeting most of the point mutations in an allele-specific manner. CrisPam simplifies the gRNAs design process to specifically target the allele of interest and scans a wide range of 26 unique PAMs derived from 23 Cas enzymes.

CrisPam is freely available at https://www.danioffenlab.com/crispam.

## Introduction

The clustered regularly interspaced short palindromic repeats (CRISPR)-Cas system enables precise genome editing mediated by the guide RNA (gRNA), an RNA molecule that directs the CRISPR associated (Cas) protein to the target DNA within the genome. The Cas enzyme, the catalytic unit of the CRISPR-Cas system, generates a DNA double-strand break in the presence of a DNA:gRNA complementary and a protospacer-adjacent motif (PAM) in immediate proximity to the target DNA (Jinek et al., 2012; Cong et al., 2013). The diversity of Cas proteins, derived from distinct bacterial strains, differ in several properties such as PAM sequence, cleavage pattern and position, sequence length, efficacy in different organisms, off-targets, and substrate (DNA or RNA). The standard Cas protein has been modified to broaden its applications to base-editing (Komor et al., 2016; Gaudelli et al., 2017), transcription repression and activation (Bikard et al., 2013; Gilbert et al., 2014; Konermann et al., 2015), epigenomic modifications (Hilton et al., 2015), visualization of genomic loci (Chen et al., 2013), and DNA nicking (Ran et al., 2013; single-strand cleavage). In an experiment design, the PAM sequence and size of the designated Cas should be taken under consideration; presence of a PAM is a limiting step in targeting unique loci, and the Cas size determines the optional delivery systems (e.g., viral vectors).

## SNP-Derived PAM

The CRISPR-Cas system can tolerate mismatches between the gRNA and the target DNA. The bases at positions 8–13 at the PAM-proximal end of the spacer (regarding type II Cas proteins) are termed the seed sequence along with the first base at the 5' end (Jinek et al., 2012; Cong et al., 2013; Hsu et al., 2013; Anderson et al., 2015; Doench et al., 2016). Mismatches within the seed sequence are thought to be intolerable and abolish DNA cleavage (Cong et al., 2013; Anderson et al., 2015). However, previous studies have shown that a single mismatch between the gRNA and the target DNA results in a non-specific knockdown of both the mutant and the wild-type alleles in some proportion (Hsu et al., 2013; Doench et al., 2016; Capon et al., 2017; Christie et al., 2017). Thus, the nature of specificity of the Cas enzyme is seemingly insufficient to specifically target single-nucleotide variations (SNVs). A single-nucleotide polymorphism (SNP)-derived PAM approach overcomes this potential limitation of targeting the disease-causing allele, while leaving the wild-type allele intact. This method dramatically increases the specificity of targeting the mutant allele alone by identifying a PAM sequence that is present only at the mutant sequence (György et al., 2019). Meaning, the SNV causes the formation of the PAM sequence, thus allows cleavage of the target allele and prevents the unintended cleavage of the reference allele (Wu et al., 2013; Courtney et al., 2016; Christie et al., 2017). Moreover, studies have shown that allele-specific targeting in heterozygous cells results in high rates of gene conversion (14–47%), where the homologous chromosome serves as a template for homologous recombination to resolve the double-strand break (Wu et al., 2013; Filler Hayut et al., 2017). In such cases, targeting the pathogenic allele results in its correction by the wild-type allele. These findings emphasize the potential of pathogenic allele-specific targeting in heterozygous patients, suffering from haploinsufficiency or gain-of-function associated diseases. Here, we discuss the utility of the SNP-derived PAM approach and use the terminology as termed by Courtney et al. (2016), although we refer to SNVs in general regardless of their frequency in the population. Most Cas proteins may be appropriate candidates for targeting a gene without a location preference of DNA cleavage, mainly used for gene-knockout experiments. However, when targeting a SNV in general, or if utilizing the SNP-derived PAM approach in particular, the selection of Cas proteins is limited. This is mostly due to the condition of PAM presence in proximity to the SNV or having a PAM generated by the SNV. Current gRNA design tools are based on reference genomes and, therefore, are not suitable for SNV targeting (Bae et al., 2014; Stemmer et al., 2015; Doench et al., 2016; Haeussler et al., 2016; Labun et al., 2016). In a recent study, AlleleAnalyzer, a bioinformatic tool was reported to target SNPs by obtaining sequences data from the 1,000 Genomes project. By utilizing disease-associated haplotypes, AlleleAnalyzer designs allele-specific dual gRNAs (Keough et al., 2019). Scott and Zhang (2017) and Lessard et al. (2017) also developed bioinformatic tools to design gRNAs that target conserved genomic loci to avoid gRNA incompatibility due to genetic variations. Furthermore, both studies emphasize the need of common gRNA designs due to regulations and its consequential costs. Another recently published web tool, SNP-CRISPR, designs gRNAs for non-reference genomes to support allelic targeting (Chen et al., 2020). SNP-CRISPR calculates the gRNA efficiency score for the variant and the reference sequences. However, it does not support SNP-derived PAM targeting, nor Cas enzymes options other than SpCas9 with varied PAM sequences to expand the targeting scope. Manual design of SNP-derived PAM-based gRNAs is challenging due to the wide repertoire of available Cas enzymes, their different PAMs and the complexity of some PAMs allowing variable bases in certain positions (e.g., NNGRRT). To that end, we established CrisPam, a web-tool that identifies Cas enzymes capable of allele-specific targeting *via* SNP-derived PAM. CrisPam does not refer to a reference genome and supports multiple input methods to facilitate simple user experience. Experimental researchers attempting to specifically target a SNV, either for development of CRISPR-based therapeutics or disease modeling, will benefit the utility of CrisPam for successful allele-specific gRNA designs.

## Materials and Methods

### Parsing and SNP Data Analysis

CrisPam is a pythonic code that performs sequence analysis of SNVs. The input of the code is the variant position (reference and variation nucleotides) and its flanking sequences (upstream and downstream). The code is available at https://github.com/RoyRabinowitz/CrisPam.

The dataset of human pathogenic SNPs was generated from dbSNP (build 153), incorporating data from UCSC Genome Browser and ClinVar, as previously described Rabinowitz et al. (2020).

### Implementation

CrisPam is webserver-based tool. Thus, no software installation effort is required. The CrisPam DB is a.xlsx file and can be opened by Excel. The CrisPam script is written in Python 3.6 and uses standard libraries.

## Results

### CrisPam

**Figure 1:**
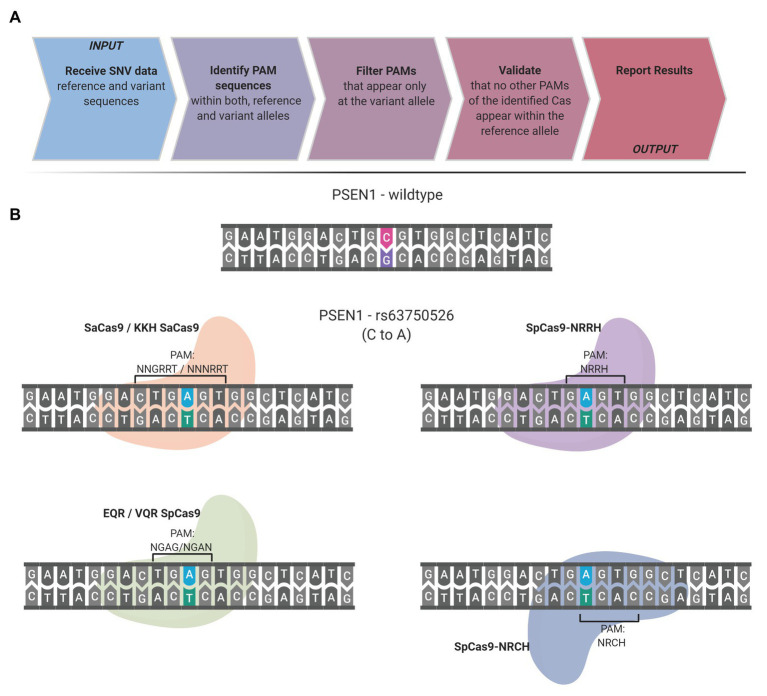
CrisPam workflow and single-nucleotide polymorphism (SNP)-derived protospacer adjacent motif (PAM) case study. **(A)** CrisPam’s analysis pipeline. **(B)** A case study: the rs63750526 SNP of the PSEN1 gene, known as a risk factor for early onset Alzheimer’s disease, as an example of multiple PAMs generated by a SNP. The base substitution (C to A) generates the PAM sequences of six Cas variants (SaCas9, KKH SaCas9, EQR Cas9, VQR Cas9, SpCas9-NRRH, and SpCas9-NRCH).

The presence of a PAM solely within the desired target allele is the guiding principle of the SNP-derived PAM methodology. The following workflow occurs to detect unique PAMs generated by a SNP: the code obtains the reference and variation bases and the flanking DNA sequences upstream and downstream to the SNV. The complementary strand is generated and analyzed to detect unique PAMs on the complementary strand as well. Some SNVs have more than a single variation nucleotide; each variation is handled individually. CrisPam attempts to detect PAMs on both the reference and the variant alleles and suggests suitable Cas variants, in case their PAM is present exclusively within the variant allele. For a given SNV, more than one PAM may be generated; therefore, CrisPam reports all the matches for the query SNV (Figure 1A). The suggested gRNA sequence for each matching Cas is the 20-23 nt upstream or downstream to the PAM, according to the Cas ortholog. The diverse PAM sequences were defined according to previous studies that characterized the unique properties and PAM compatibility of each Cas. Several Cas enzymes recognize multiple sequences as legitimate PAMs (e.g., enAsCas12a recognizes TGTV, VTTV, TTTT, and TTCN). For a hypothetical point mutation TT<C>TA to TT<T>TA (C to T), enCas12a might be erroneously suggested as a possible candidate Cas since the point mutation generates its TTTT PAM. However, the reference allele contains an alternative PAM – TTCN of the same Cas; therefore, an unintended targeting of the reference allele will presumably occur. For multiple-PAMs Cas enzymes, CrisPam validates that the reference sequence is not targetable by any PAM of the suggested Cas. The well-studied SpCas9 is known to be recognizing mainly NGG PAMs; however, it may exhibit some degree of binding to target DNA sequences with NAG PAMs (Hsu et al., 2013). While CrisPam considers SpCas9 as a potential candidate only when the NGG PAM is generated by the variant allele, NAG is defined as a potential off-target PAM. Therefore, SpCas9 would not be identified as a possible match in case the NAG PAM is present within the reference sequence. Currently, CrisPam identifies 26 PAM sequences of 23 Cas enzymes (Table 1).

**Table 1:** Cas diversity and properties.

Type	Cas	PAM sequence	Size (bp)	PAM orientation
II	SpCas9 (Jinek et al., 2012)	NGG (NAG)	≈4,100	Downstream of the target DNA
	VRER SpCas9 (Kleinstiver et al., 2015b)	NGCG		
	EQR SpCas9 (Kleinstiver et al., 2015b)	NGAG		
	VQR SpCas9 (Kleinstiver et al., 2015b)	NGAN		
	SpCas9-NG (Nishimasu et al., 2018)	NG, NANG		
	xCas9 (Hu et al., 2018)	NG		
	SpCas9-NRRH (Miller et al., 2020)	NRRH, NGGN		
	SpCas9-NRTH (Miller et al., 2020)	NRTH, NGGN		
	SpCas9-NRCH (Miller et al., 2020)	NRCH, NGGN		
	SaCas9 (Ran et al., 2015)	NNGRRT	≈3,150	
	KKH SaCas9 (Kleinstiver et al., 2015a)	NNNRRT		
	NmCas9 (Esvelt et al., 2013)	NNNNGATT	≈3,240	
	St1Cas9 (Xu et al., 2015; Agudelo et al., 2020)	NNRGAAW	≈4,180	
	St3Cas9 (Magadán et al., 2012)	NGGNG		
	TdCas9 (Sun et al., 2018)	NAAAAC	≈4,200	
	CjCas9 (Kim et al., 2017)	NNNVRYM	≈2,950	
	SpCas9 (from *Streptococcus pasteurianus*; Tan et al., 2018)	NNGTGA	≈3,400	
V	AsCas12a and LbCas12a (Zetsche et al., 2015)	TTTN	≈3,685	Upstream of the target DNA
	AsCas12a RVR (Gao et al., 2017)	TATV		
	AsCas12a RR (Gao et al., 2017)	TYCV		
	enAsCas12a (Kleinstiver et al., 2019)	TGTV, VTTV, TTTT, TTCN		
	FnCas12a (Fonfara et al., 2016)	TTV	≈3,900	
	Cas12e (CasX; Burstein et al., 2017)	TTCN	≈2,940	

### A Database of PAM-Generating SNPs

**Figure 2:**
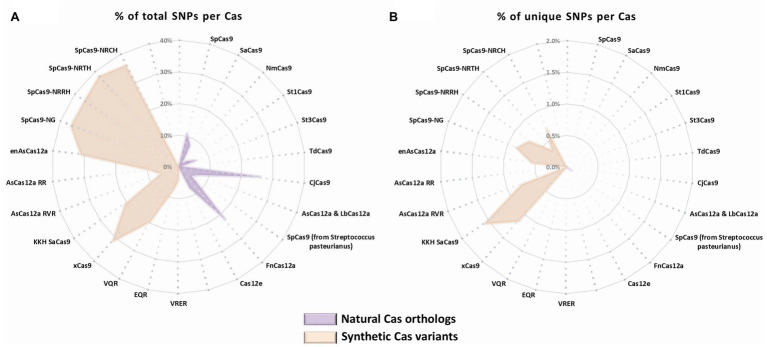
SNPs matching coverage comparison by Cas. Cas natural orthologs (purple) and synthetic variants (orange) representation of **(A)** total matching SNPs for each Cas (% out of total SNPs) and **(B)** exclusive matches of SNPs for each Cas variant (% out of total SNPs).

To assess the potential of the SNP-derived PAM approach to treat human pathogenic point mutations, we used CrisPam to analyze a total of 43,673 SNPs (pathogenic and likely-pathogenic SNPs) from dbSNP (Sherry et al., 1999). Successful matches of SNPs that generate at least a single PAM were found in 90% of the total SNPs (39,307 out of 43,673). Figure 1B represents a case study of a SNP (rs63750526 of the PSEN1 gene) that generates six PAMs. Such multiple-PAM generating SNPs confer the ability to opt the most suitable Cas depending on the application limitations such as vector size and on-target efficiency. We sought to assess the importance of synthetic Cas variants and their additive value to the variety of Cas enzymes. To that end, we compared the SNPs matching coverage of each Cas based on our analysis over the pathogenic SNPs dataset (Figure 2). Notably, synthetic variants account for a large proportion of the successful matches. In 6.65% of the analyzed SNPs (2,907 out of 43,673), a synthetic variant was reported to be solely Cas that is able to target the SNP in a SNP-derived PAM manner (Figure 2B). The full database (DB) of PAM-generating pathogenic and likely pathogenic SNPs is available as a Supplementary Material (Supplementary Material S1). Analyzing the data reveals that the 10% SNPs that were not targetable, share a certain type of base substitution: C→T or G→A (Supplementary Material S2). This can be rationalized by the abundance of G-rich and relaxed PAMs, compared to T-rich PAMs that tend to require more rigorous sequences.

### CrisPam – An Online SNP-Derived PAM Analysis Tool

We established a web tool that performs CrisPam’s SNP-derived PAM targeting abilities on user data. Multiple input methods are available to allow analysis of SNVs that are yet to be reported and included in NCBI’s dbSNP or non-pathogenic SNVs for research purposes. SNV data are accepted in one of the following methods: user manual input, fetch SNV data by rsID, fetch SNV by genomic coordinates (from 56 genomes), and a batch file that allows analysis of multiple SNVs. User-defined PAM is supported to enable researchers to identify suitable Cas proteins that are yet to be reported. The CrisPam web tool also supports single-nucleotide insertion and deletion variations. For NGG-based Cas variants, off-targets assessment is available *via* CRISTA (Abadi et al., 2017).

CrisPam is available at https://www.danioffenlab.com/crispam.

## Discussion

The SNP-derived PAM targeting approach for enhancing allele specificity is a promising method for future CRISPR-based novel genome-editing therapies. As many patients suffering from genetic conditions are heterozygous, carrying one copy of a pathogenic allele, it is essential to develop SNP customized treatments for increasing treatment’s safety. Currently, CrisPam supports off-targets assessment only for NGG-based Cas enzymes. For non-NGG enzymes, it is suggested to use complementary tools to assess potential off-targets (Stemmer et al., 2015; Abadi et al., 2017; Hwang et al., 2018). We recommend using CrisPam as the first step in the pipeline of gRNA design. For multiple-PAM generating SNPs, considerations such as delivery vector capacity [e.g., adeno-associated virus (AAV) or lentivirus] and efficiency may also define the most suitable Cas enzyme for the experiment. The webtool is ideal for researchers that intend to specifically target certain variations, while it may be less effective as a starting point for identifying target SNPs. Though, the CrisPam DB presents targetable SNPs and the relevant Cas enzymes to perform allele-specific targeting. Not all SNPs’ clinical significance is commonly agreed or yet to be known and such SNPs may not be included in the dataset we used to generate the CrisPam DB. For that reason, it is suggested to use the CrisPam webtool if the SNP of interest does not appear in the CrisPam DB. Previous studies reported that genetic variations, in rare cases, may change the genomic target DNA and form mismatches between the target DNA and the gRNA or even disrupt the PAM site. Not only that genetic variations influence on-target activity, they may also cause altered potential off-targets sites. Therefore, for clinical purposes, it is essential to perform whole-genome sequencing for patients and validate on-target sequence integrity and detect potential personalized off-targets (Lessard et al., 2017; Scott and Zhang, 2017).

By utilizing CrisPam, we demonstrate the high compatibility of the SNP-derived PAM approach to preform allele-specific targeting using varied CRISPR-Cas systems. As demonstrated on human pathogenic SNPs data, 90% of the SNPs were found to have a SNP-derived PAM. Our findings underline the relevance of this allele-specific targeting approach in developing genome-editing therapeutic strategies. While CRISPR applications have been widely expanded, the SNP-derived PAM approach may be utilized for gene silencing (using inactive Cas), genetic screening, and more applications other than allele-specific DNA cleavage. This study emphasizes the emerging importance of broadening PAM compatibility of Cas proteins to enable allele-specific targeting and overcome the PAM limitation. Furthermore, CrisPam offers a simple interface to design an allele-specific targeting experiment using the CRISPR-Cas system.

## Data Availability Statement

Project home page: https://www.danioffenlab.com/crispam. Programming language: Python 3.6. The script is available at https://github.com/RoyRabinowitz/CrisPam. The SNPs dataset that was used to generate the CrisPam DB for PAM-generating pathogenic \ likely-pathogenic SNPs was previously described in “Prediction of Synonymous Corrections by the BE-FF Computational Tool Expands the Targeting Scope of Base Editing” (Rabinowitz et al. 2020). The script is available at https://github.com/shiranab/parse_dbSNP. The obtained dataset of SNP-derived PAM targetable SNPs is available at the supplementary files (**sup. file S1**). License: Free for academic end-users solely for non-commercial research purposes. Any restrictions to use by non-academics: Contact Prof. Dani Offen or Ramot at Tel Aviv University LTD. The datasets presented in this study can be found in online repositories.

## Author Contributions

Conceived and designed the study, analyzed the data, and wrote the manuscript: RR. Programing: SA, RD, and RR. Webserver and user interface integration: SA. Principle investigator: DO. All authors contributed to the article and approved the submitted version.

### Conflict of Interest

The authors declare that the research was conducted in the absence of any commercial or financial relationships that could be construed as a potential conflict of interest.
